# A vessel segmentation method for multi-modality angiographic images based on multi-scale filtering and statistical models

**DOI:** 10.1186/s12938-016-0241-7

**Published:** 2016-11-08

**Authors:** Pei Lu, Jun Xia, Zhicheng Li, Jing Xiong, Jian Yang, Shoujun Zhou, Lei Wang, Mingyang Chen, Cheng Wang

**Affiliations:** 1 Research Centre for Medical Robotics and Minimally Invasive Surgical Devices, Institute of Biomedical and Health Engineering, Shenzhen Institutes of Advanced Technology, Chinese Academy of Sciences, Shenzhen, 518055 China; 2 Radiology Department, Shenzhen Second People’s Hospital, The First Affiliated Hospital of Shenzhen University, Shenzhen, 518035 China; 3 Beijing Engineering Research Centre of Mixed Reality and Advanced Display, School of Optics and Electronics, Beijing Institute of Technology, Beijing, 100081 China

**Keywords:** Multi-modality angiographic images, Vessel segmentation, Multi-scale filtering, Statistical mixture model, Markov random field

## Abstract

**Background:**

Accurate segmentation of blood vessels plays an important role in the computer-aided diagnosis and interventional treatment of vascular diseases. The statistical method is an important component of effective vessel segmentation; however, several limitations discourage the segmentation effect, i.e., dependence of the image modality, uneven contrast media, bias field, and overlapping intensity distribution of the object and background. In addition, the mixture models of the statistical methods are constructed relaying on the characteristics of the image histograms. Thus, it is a challenging issue for the traditional methods to be available in vessel segmentation from multi-modality angiographic images.

**Methods:**

To overcome these limitations, a flexible segmentation method with a fixed mixture model has been proposed for various angiography modalities. Our method mainly consists of three parts. Firstly, multi-scale filtering algorithm was used on the original images to enhance vessels and suppress noises. As a result, the filtered data achieved a new statistical characteristic. Secondly, a mixture model formed by three probabilistic distributions (two Exponential distributions and one Gaussian distribution) was built to fit the histogram curve of the filtered data, where the expectation maximization (EM) algorithm was used for parameters estimation. Finally, three-dimensional (3D) Markov random field (MRF) were employed to improve the accuracy of pixel-wise classification and posterior probability estimation. To quantitatively evaluate the performance of the proposed method, two phantoms simulating blood vessels with different tubular structures and noises have been devised. Meanwhile, four clinical angiographic data sets from different human organs have been used to qualitatively validate the method. To further test the performance, comparison tests between the proposed method and the traditional ones have been conducted on two different brain magnetic resonance angiography (MRA) data sets.

**Results:**

The results of the phantoms were satisfying, e.g., the noise was greatly suppressed, the percentages of the misclassified voxels, i.e., the segmentation error ratios, were no more than 0.3%, and the Dice similarity coefficients (DSCs) were above 94%. According to the opinions of clinical vascular specialists, the vessels in various data sets were extracted with high accuracy since complete vessel trees were extracted while lesser non-vessels and background were falsely classified as vessel. In the comparison experiments, the proposed method showed its superiority in accuracy and robustness for extracting vascular structures from multi-modality angiographic images with complicated background noises.

**Conclusions:**

The experimental results demonstrated that our proposed method was available for various angiographic data. The main reason was that the constructed mixture probability model could unitarily classify vessel object from the multi-scale filtered data of various angiography images. The advantages of the proposed method lie in the following aspects: firstly, it can extract the vessels with poor angiography quality, since the multi-scale filtering algorithm can improve the vessel intensity in the circumstance such as uneven contrast media and bias field; secondly, it performed well for extracting the vessels in multi-modality angiographic images despite various signal-noises; and thirdly, it was implemented with better accuracy, and robustness than the traditional methods. Generally, these traits declare that the proposed method would have significant clinical application.

## Background

Nowadays, cardio- and cerebro-vascular diseases have greatly threatened human health. Since the use of imaging techniques such as computed tomography angiography (CTA) and magnetic resonance angiography (MRA) in minimally invasive surgery, high quality image segmentation has become an important area of interest. A detailed review has been made on threshold based, pattern recognition based, and deformable models based segmentation algorithms which were used for medical images [1, 2]. The main tendency of these algorithms with their principle ideas, application field, advantages and disadvantages were discussed. Conclusion has been drawn that each segmentation method with improvement, or in combination with other technique, could provide better performance [3, 4]. Clustering algorithms and supervised classification used for the segmentation of atherosclerotic plaques were analysed in [5].

For the diagnosis and treatment of vascular diseases, it is critical to accurately extract and quantify the blood vessels from the angiographic image. Aimed at the extraction of blood vessels, different kinds of segmentation methods have been proposed, e.g.: multi-scale filtering [6–9], deformable models [10], statistical models [11–13], and hybrid methods [14, 15]. Multi-scale filtering can enhance the intensity of the vessels while suppresses that of the background [16] in various angiographic data, however, further processing should be conducted in order to label the vessel class out. Deformable models well integrate bottom–up information and top–down priori knowledge, but the segmentation quality mainly depends on the model parameters [17, 18]. For statistical models, the vessels in the angiographic data with a given modality are segmented according to the intensity distributions of anatomical structures, by which voxels with overlapped intensities will inevitably be misclassified [19]. In order to reduce the probability of error classification, hybrid methods have been proposed to take the spatial contextual information into account [20], but limitations still exist for multi-modality angiographic images.

So far, statistical models have drawn a lot of attention, and model selection is an important issue in this kind of vessel segmentation techniques. To our known, the earliest statistical-based segmentation algorithm for extracting 3D cerebral vessels from MRA data was established by Wilson and Noble [19]. They divided the MRA data histogram into three regions, modelled by a mixture model of two Gaussian distributions and one uniform distribution. Hassouna et al. [21] improved the mixture model with a Rayleigh distribution and three Gaussian distributions to provide an accurate fitting of the histogram curve. On the basis of statistical model analysis and improved curve evolution, a fast segmentation algorithm for extracting the 3D cerebral vessels was presented by Gao et al. [22]. To segment fine cerebral vessels with complicated contexts, a statistical method based on maximum a posteriori and Markov random field (MAP–MRF) with multi-pattern neighbourhood system (MP-NBS) was proposed by Zhou et al. [23], where the larger cerebrovascular network can be extracted out automatically. In general, statistical segmentation methods need to construct mixture probability distributions and adjust their parameters to fit a certain image histogram, where the vessels in a particular position are segmented according to the specific intensity distribution of a certain angiography image. To sum up, a given statistical model only deals with a specific imaging modality on a given anatomical part, which is to say that the statistical segmentation method should change its models to fit a certain medical data. This issue has greatly restricted the application of statistical methods.

Being different from the above-mentioned statistical vessel segmentation method, in this paper, a flexible method for extracting vessels from various modalities of angiographic images was developed. Up to present, statistical modelling against the data of multi-scale filtering response was not been used to vessel segmentation from angiographic data. One reason lies in the complexity of constructing mixture model, and the other lies in the error being not easily controlled with parameters estimation. We have done this attempt, and our method includes the following several steps. In the first step, image data of different modalities were filtered by multi-scale vessel enhancement algorithm. The intensity of the vessels was enhanced, whereas the intensity of the background voxels was suppressed, and the filtered data turned to a new distribution. Then, the filtered data was modelled by a new mixture model consisting of reduced distribution classes. Based on the histogram curve analysis, a Gaussian distribution was used to model the high intensity which represents the vessels; while two Exponential distributions were used to model the low and middle intensity regions which represent the background voxels. The parameters of the mixture model were estimated by the EM algorithm. At last, the vessels were marked out by MAP–MRF algorithm.

The rest of this paper is organized as follows. In “Methods” section, the proposed method is described in detail, where the multi-scale filtering based vessel enhancement, histogram analysis, the mixture model constructing for histogram curve fitting, parameters estimation, and the MAP–MRF segmentation algorithm are presented in succession. “Experiments” section details the experiments that were conducted on a series of phantoms, the MRA data, and the CTA data, where results are also presented. Summaries and future work are discussed in “Results and discussions” section, and the conclusions are drawing in the last section.

## Methods

**Fig. 1:**
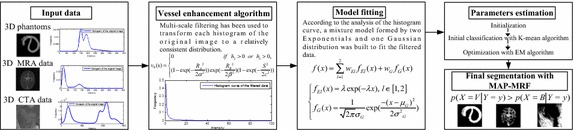
The framework of the proposed segmentation method

The framework of the proposed method is shown in Fig. 1. Firstly, to attain a relatively consistent histogram characteristic from multi-modality input data, multi-scale filtering is employed as the pre-processing step. Secondly, a mixture model composed of three distributions was built to fit the histogram curve of the filtered data. The parameters of the three distributions were automatically estimated through peak-points detecting of the histogram curve, K-means clustering algorithm, and EM algorithm. Finally, the blood vessels were segmented by the MAP–MRF algorithm.

### Vessel enhancement algorithm

We use the algorithm of three dimensional multi-scale filtering algorithm [6] to strengthen the intensity of the vessels while weakening that of the background. According to the eigenvalues of Hessian, the vessel function with multi-scale filtering is expressed as:

1 $$v_{0} ({\rm s}) = \left\{ \begin{array}{l} 0\quad if\;\;h_{2} > 0\;\;or\;\;h_{3} > 0, \\ \left(1 - \exp \left( - \frac{{R_{A}^{2} }}{{2\alpha^{2} }}\right)\right)\exp \left( - \frac{{R_{B}^{2} }}{{2\beta^{2} }}\right)\left(1 - \exp \left( - \frac{{S^{2} }}{{2c^{2} }}\right)\right)\end{array} \right.$$

where *R*_*A*_, *R*_*B*_ and *S* are the three measures that can be respectively controlled by the parameters *α*, *β* and *c*, so that the vessel function be sensitive to the vessel shapes. The relationships between these parameters and the eigenvalues are shown in the following:

2 $$\left\{ \begin{array}{lll} R_{A} = \frac{{\left| {h_{2} } \right|}}{{\left| {h_{3} } \right|}}\\ R_{B} = \frac{{\left| {h_{1} } \right|}}{{\sqrt {\left| {h_{2} h_{3} } \right|} }} \\ S = \left\| H \right\|_{F} = \sqrt {\sum\nolimits_{j \le D} {h_{j}^{2} } } \\ \end{array} \right.$$

Here, *h*_1_, *h*_2_, and *h*_3_ are the eigenvalues corresponding to the eigenvectors of three orthonormal directions which are robust to the scaling factor. The resultant ratios *R*_*A*_ and *R*_*B*_ would be sensitive to the shapes of the blood vessel structure (e.g., blob-, plate-, and line-like structures), while the measure *S* would be used to suppress image noise.

**Fig. 2:**
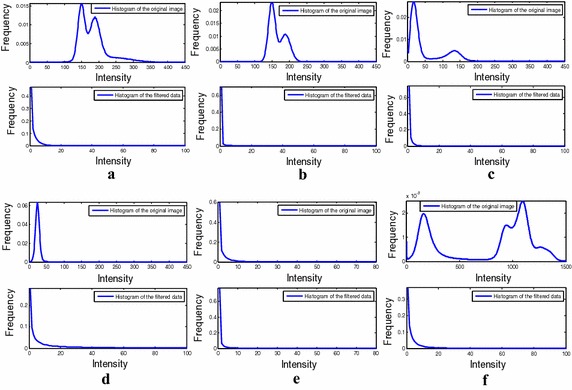
The histogram curves of both the original data and the filtered data from **a** to **b** phantoms **c**–**e** MRA, and **f** CTA

The above-mentioned enhancement algorithm was tested on several angiographic data sets. The histogram curves of the original data sets and those of the corresponding filtered data sets are compared in Fig. 2. Note that the low, middle, and high intensity regions of the filtered data gained from various modality original data exhibit similar distribution features.

After processed using multi-scale filtering, the vessels would be enhanced while the background tissues would be weakened, such that the resultant histogram curve of the filtered data would be fitted by reduced model elements.

### Mixture model construction

Through the comparison of the histogram curves between the original and the filtered data in Fig. 2, it can be seen obviously that the vessels occupy the high intensities region with a range of gradual change, thus a Gaussian distribution could be used to model the vessel class. For the background class, the Exponential distributions showed the best fitting performance after several probability distribution functions were tested. Therefore, two Exponential distributions were used to model the low and middle intensity regions of background voxels. As a result, the mixture model for the filtered data can be described as a linear combination of the three probability distribution functions:

3 $$f(x) = \sum\limits_{l = 1}^{2} {w_{El} f_{El} (x)} + w_{G} f_{G} (x)$$

where the mixture model *f*(*x*) is composed of Exponential distributions *f*_*E*1_(*x*) and *f*_*E*2_(*x*) as well as Gaussian distribution *f*_*G*_(*x*). The parameters *w*_*E*1_, *w*_*E*2_, and *w*_*G*_ are the proportion coefficients whose sum is unity. The three distributions can be defined as follows:

4 $$f_{El} (x) = \lambda \exp ( - \lambda x), l \in \left[ {1,2} \right]$$

5 $$f_{G} (x) = \frac{1}{{\sqrt {2\pi } \sigma_{G} }}\exp \left(\frac{{ - (x - \mu_{G} )^{2} }}{{2\sigma^{2}_{G} }}\right)$$

where parameters *λ*, *μ*, *σ*, and the proportion coefficients *w*_*El*_, *w*_*G*_ should be firstly estimated before the classification procedure. To begin with, the intensity range of the filtered data was enlarged to be consistent with the original image data. Then, the filtered data was classified into three parts using the K-means algorithm. For the initialization of the K-means algorithm, the histogram curve of the original image was smoothed, and the peak points were automatically found out through local extremum method. If there were more than three peak points in the original histogram curve, the K-means algorithm would be initialized by the first three peak values. Otherwise, it would be initialized according to the following rule:

6 $$\left\{ \begin{array}{l} \mu_{k\_1}^{init} = I_{\text{max} } \\ \mu_{k\_2}^{init} = (I_{\text{max} } + I_{\text{min} } )/2 \hfill \\ \mu_{k\_3}^{init} = I_{\text{min} } \\ \end{array} \right.$$

where *I*_max_ and *I*_min_ are the maximum and the minimum intensity values of the filtered data. The variables $\mu_{k\_1}^{init}$, $\mu_{k\_2}^{init}$, and $\mu_{k\_3}^{init}$ are the initial cluster centroids while *μ*_*k*_*l*_, *σ*_*k*_*l*_, *w*_*k*_*l*_,  (*l* = 1, 2, 3) represent the distribution parameters which were all estimated by the K-means algorithm.

As the accuracy of theses parameters plays an important role in the final classification, the EM algorithm was used to improve the estimation performances of these parameters. The results of the K-means algorithm were used as the initial values for the EM algorithm, as shown in Eq. (7):

7 $$\left\{ \begin{array}{l} \lambda_{E1}^{init} = 1.0/\mu_{k\_1} ,w_{E1}^{init} = w_{k\_1} \\ \lambda_{E2}^{init} = 1.0/\mu_{k\_2} ,w_{E2}^{init} = w_{k\_2} \\ \mu_{G}^{init} = \mu_{k\_3} ,\sigma_{G}^{init} = \sigma_{k\_3} ,w_{G}^{init} = w_{k\_3} \\ \end{array} \right.$$

Then, the parameters were iteratively updated with the EM algorithm as follows:

8 $$\left\{ {\begin{array}{l} {\lambda_{El}^{k + 1} = \frac{{\sum\nolimits_{j = 1}^{N} {f^{k} (El|x_{j} )} }}{{\sum\nolimits_{j = 1}^{N} {f^{k} (El|x_{j} )x_{j} } }}} \\ {w_{El}^{k + 1} = \frac{1}{N}\sum\nolimits_{j = 1}^{N} {f^{k} (El|x_{j} )} } \\ \end{array} } \right.,l \in \left[ {1,2} \right]$$

9 $$\left\{ \begin{array}{l} \mu_{G}^{k + 1} = \frac{{\sum\nolimits_{j = 1}^{N} {f^{k} (G|x_{j} )x_{j} } }}{{\sum\nolimits_{j = 1}^{N} {f^{k} (G|x_{j} )} }},w_{G}^{k + 1} = \frac{1}{N}\sum\nolimits_{j = 1}^{N} {f^{k} (G|x_{j} )}\\ (\sigma_{G}^{k + 1} )^{2} = \frac{{\sum\nolimits_{j = 1}^{N} {f^{k} (G|x_{j} )} (x_{j} - \mu_{G}^{k + 1} )^{2} }}{{\sum\nolimits_{j = 1}^{N} {f^{k} (G|x_{j} )} }} \\ \end{array} \right.$$

where N is the total number of the voxels in the filtered data and *x*_*j*_ is the corresponding intensity of voxel *j*. The posterior probability *f* ^*k*^(*El*|*x*_*j*_) and *f* ^*k*^(*G*|*x*_*j*_) are gained by the Bayes formula that are explained in Eq. (10)

10 $$\left\{ {\begin{array}{*{20}c} {f^{k} (El|x_{j} ) = \frac{{w_{{_{El} }}^{k} f^{k} (x_{j} |El)}}{{\sum\nolimits_{l = 1}^{2} {w_{{_{El} }}^{k} f^{k} (x_{j} |El)} \,+\, w_{{_{G} }}^{k} f^{k} (x_{j} |G)}}} \\ {f^{k} (G|x_{j} ) = \frac{{w_{{_{G} }}^{k} f^{k} (x_{j} |G)}}{{\sum\nolimits_{l = 1}^{2} {w_{{_{El} }}^{k} f^{k} (x_{j} |El)} \,+\, w_{{_{G} }}^{k} f^{k} (x_{j} |G)}}} \\ \end{array} } \right.$$

### MAP–MRF based classification

A series of filtered data was modelled by the proposed mixture model, and the combination of two Exponential distributions and one Gaussian distribution shows a good fitting to the histogram curves of the filtered data from the multi-modality angiographic images. According to the MAP algorithm, a voxel belongs to the vessel class only if it meets the following criteria:

11 $$w_{G} f_{G} (x_{j} ) > w_{E1} f_{E1} (x_{j} ) + w_{E2} f_{E2} (x_{j} )$$

Equation (11) gives the simplest case of binary vessel labelling, in which the voxels of the filtered data are labelled as either blood vessels or background according to their posteriori probability. However, misclassification still exists when significant vascular signal losses or the noise has high intensity value because MAP classification only depends on the voxel intensity. Therefore, MRF algorithm is introduced for enhanced segmentation.

Spatial interaction between neighbouring voxels is modelled by MRF. Assume that *X* is an MRF, observed vector *Y* is conditionally independent, then MAP–MRF based expressions can be written as

12 $$p(\left. X \right|Y) \propto p(\left. Y \right|X)p(X)$$

where *p*(*X*|*Y*), being the low level MRF process, is modelled as a mixture model talked above; the term *p*(*X*), being the high level MRF process, is given by the Gibbs distribution [24] as follows

13 $$p(X = x) = \frac{\exp ( - U(x))}{Z},\quad Z = \sum\limits_{x \in \varOmega } {\exp ( - U(x))}$$

where *U*(*x*) is the energy function, *Ω* denotes the space of all possible labelling, and *Z* is a normalizing constant to be the sum of the numerator over all possible configurations of *x*. With the isotropic multi-level logistic (MLL) model [25], *U*(*x*) can be expressed as

14 $$U(x_{s} ) = \sum\limits_{r \in \eta } {\beta_{sr} V(x_{s} ,x_{r} )} \quad \forall r \in \eta$$

where *η* denotes the neighbourhood cube with the size of $3 \times 3 \times 3$, $V(x_{s} ,x_{r} )$ is the energy of pair-points in this MP-NBS [23], and the energy function *U*(*x*_*s*_) is proportional to the summation of the weighted $V(x_{s} ,x_{r} )$ with respect to *β*_*sr*_. The instances of $V(x_{s} ,x_{r})$ used are given below.

15 $$V\text{(}x_{s} ,x_{r} \text{)} = \left\{ \begin{array}{l} 1, \quad if\;\;x_{s} = x_{r} \hfill \\ 0, \quad \text{elsewhere} \end{array} \right.$$

The regularization parameter *β*_*sr*_ is meant for the strength of the interaction between pair-wise neighbouring voxels.

Overall, according to the MAP–MRF approach, the posterior probability of the vessel class can be expressed by

16 $$p(\left. {X = V} \right|Y = y) \propto f_{G} (y)\exp ( - U(V))$$

Meanwhile, the posterior probability of the background class is given by

17 $$p(\left. {X = B} \right|Y = y) \propto \frac{{w_{E1} f_{E1} (y) + w_{E2} f_{E2} (y)}}{{w_{E1} + w_{E2} }}\exp ( - U(B))$$

Finally, the MAP–MRF model is solved by iteratively using the iterated conditional modes (ICM) [26], and a voxel belongs to the vessel class only if it meets the following rule:

18 $$p(\left. {X = V} \right|Y = y) > p(\left. {X = B} \right|Y = y)$$

## Experiments

The experiment results of the proposed segmentation method are presented in this section. In order to quantitatively and qualitatively validate the proposed method, tests were conducted on phantoms and various modality angiographic images from MRA, and CTA data. Additionally, comparisons with the traditional methods have also been discussed at the end of this section.

### Experiment of quantitative validation

Since it was not easy to acquire the golden standard of vessels in real clinical data, phantoms with known ground truth and complicated background noises were designed to quantitatively validate the proposed method. The percentage of the number of misclassified voxels to that of the total voxels of the phantom was calculated as the segmentation error ratio. As a popular metric for evaluating the accuracy of automated or semi-automated segmentation methods, the Dice similarity coefficients (DSCs) was used to estimate the segmentation performance on the phantom data by comparing their results to the ground truth.

**Fig. 3:**
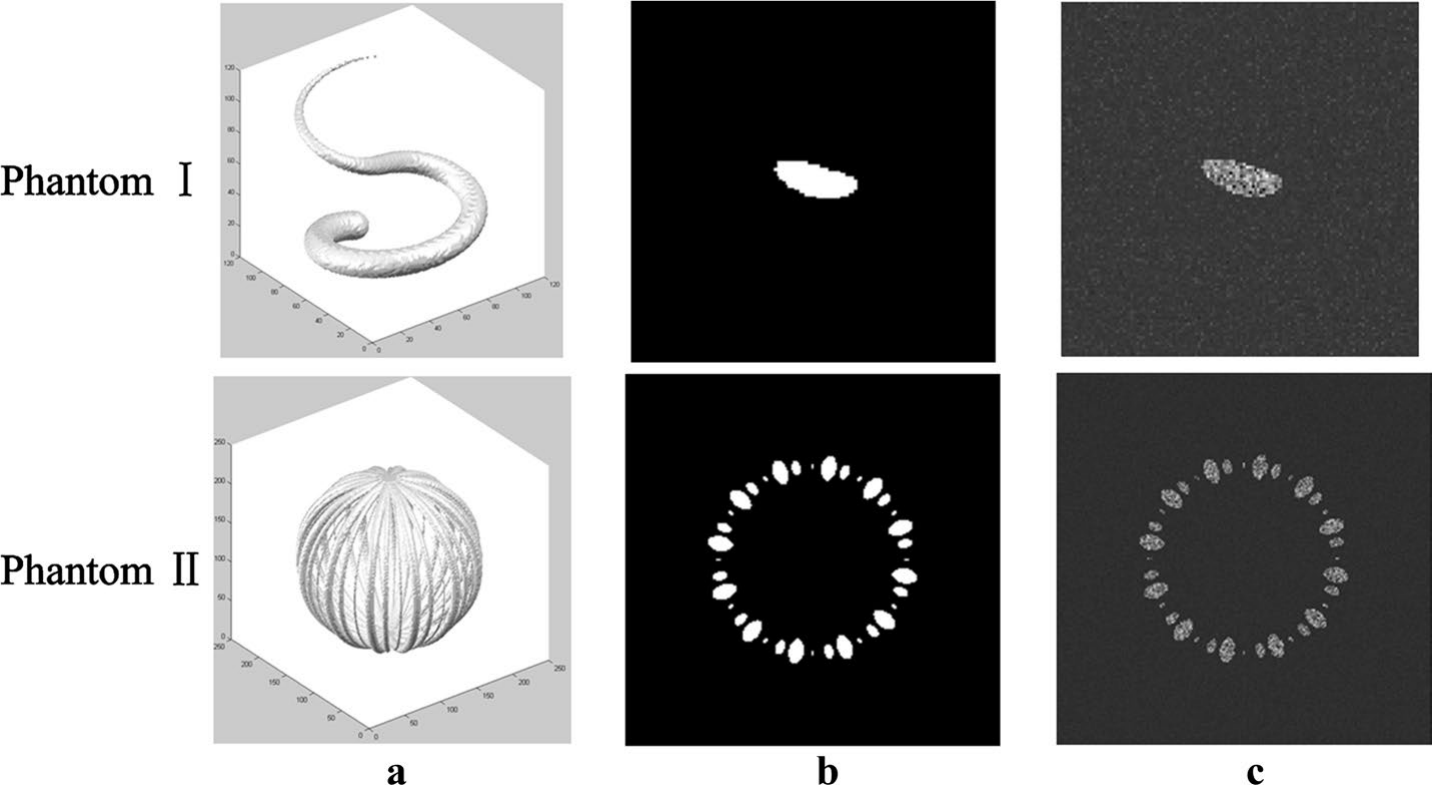
Phantoms illustration. **a** Ground truth. **b** Slices of the ground truth. **c** Phantom slices with the noises added to the ground truth

Two 3D phantoms were built to simulate the real clinical vessels data, which were shown in Fig. 3.

The simulation processes of mixture distributions for target and background were referred to our approach by Zhou et al. [23]. The main characteristics lied in follows: (1) Considering that the components of the background voxels in the angiographic images are often Gauss signals, different levels of Gaussian noises w.r.t. $(\mu ,\sigma^{2} ,\omega )$ were added into the two phantoms. (2) The proportion of the ground truth is not limited in our method, i.e., the proportions of the target and the background could be randomly set by adjusting the size of their volumes. (3) The vessels occupy the high intensities region, thus, in the setting of parameters $\text{(}\mu ,\sigma^{2} \text{, }\omega \text{)}$, the value *μ* of the vessel target should be greater than that of each Gauss noise.

Among the two phantoms one was a single curving tubular target, and the other consists of a series of tubular targets, where the intensity values of the two vessel objects were generated with stochastic ones of 810 and 650 respectively. The diameter of each tubular target smoothly changed, like that of a real vessel structure. Three Gaussian noises with the parameters $\text{(}\mu ,\sigma^{2} ,\omega \text{)}$ designed as $(148,10^{2} ,0.4)$, $(187,15^{2} ,0.4)$, and $(228,60^{2} ,0.24)$ were added into the first phantom while two Gaussian noises with the parameters $\text{(}148,10^{2} \text{, 0}\text{.5)}$, and $(187,15^{2} ,0.3)$ were added into the second phantom. The data volumes in the two phantoms were 128 × 128 × 128 and 240 × 240 × 240 voxels respectively. The 3D view of the ground truth and some slices of the phantoms were shown in Fig. 3.

**Fig. 4:**
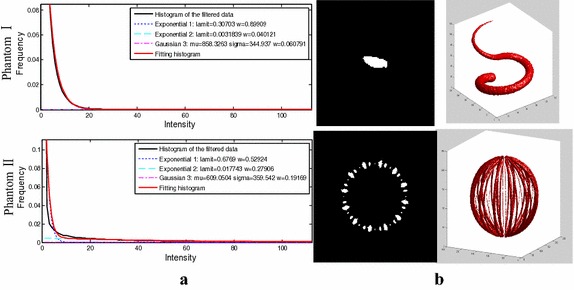
Experiments on phantoms. **a** The estimated two Exponential distributions and one Gaussian distribution curves together with their fitting curves. **b** The segmentation results with their slices and 3D views

In the first step, the phantoms were pre-processed with the multi-scale filtering algorithm. After that, the vessels were marked out using the proposed segmentation method. For the filtered data of the phantoms, the estimated two Exponential distributions and one Gaussian distribution curves were presented in Fig. 4a together with their fitting curves. Slices of the segmentation results and their 3D views were plotted in Fig. 4b. Meanwhile, the segmentation error ratios and the DSCs of the two phantoms were listed in Table 1, where *N*_*total*_ represents the total voxel number of the phantom, *N*_*over*_, and *N*_*under*_ are the number of voxels in over segmentation and under segmentation respectively. Thus, the sum of *N*_*over*_ and *N*_*under*_ is the number of voxels in misclassification. *S*_*g*_ is the set of pixels assigned to the targets in the ground truth, *S*_*m*_ is the set of pixels assigned to the targets in the segmentation result, and $N( \cdot )$ represents the number of pixels in the corresponding region. The DSC provided a measure of how accurate a method was, with its value closer to 1 indicating greater accuracy.

**Table 1:** Fitting error ratios and the DSCs for the phantoms

	Phantoms I	Phantoms II
Fitting error $\left(\frac{{N_{over} + N_{under} }}{{N_{total} }}\right)$ (%)	0.21	0.11
DSC $\left( \frac{{2N(S_{g} \cap S_{m} )}}{{N(S_{g} ) \cup N(S_{m} )}} \right)$ (%)	94.74	96.94

### Experiment of clinical validation

**Fig. 5:**
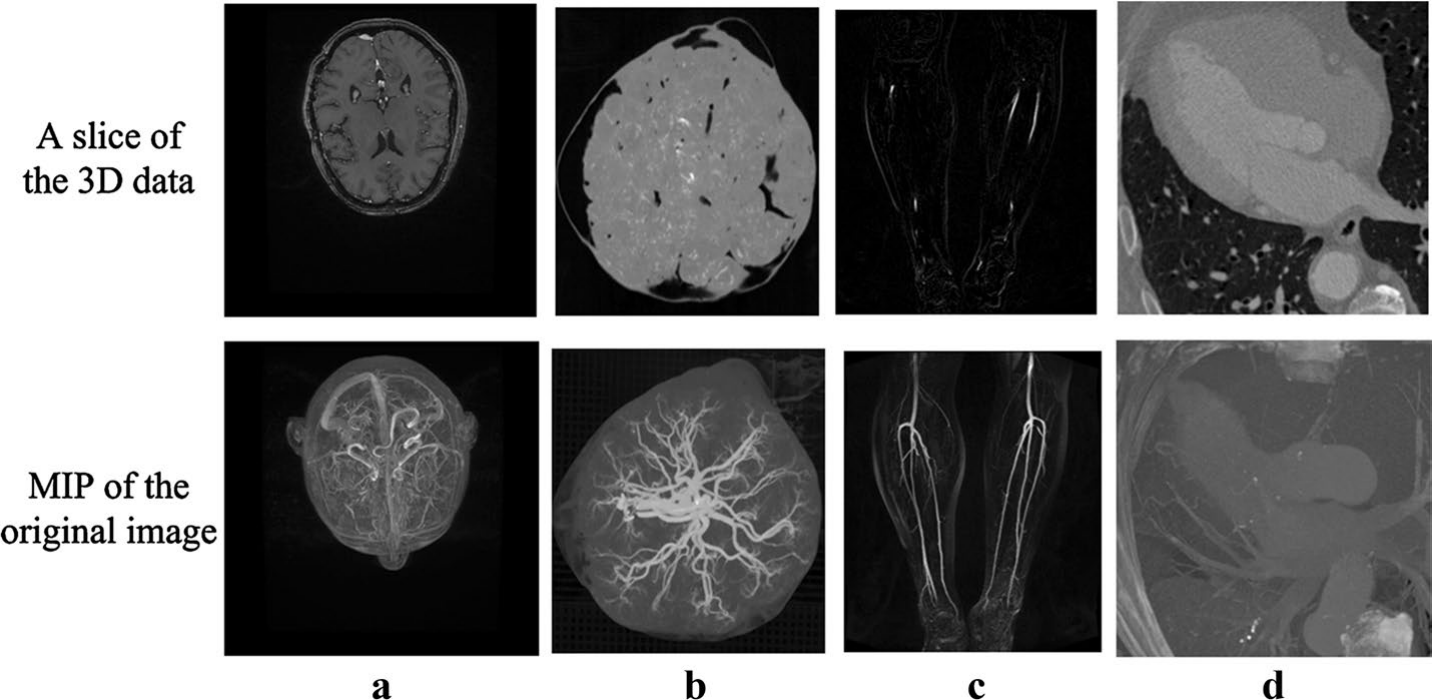
The clinical data sets. **a** Brain MRA data. **b** Placenta MRA data. **c** Legs MRA data. **d** Thorax CTA data

This section detailed the qualitative validation that was conducted on four clinical data, each of which held a different statistical characteristic. Among the four clinical data, the brain MRA data set was offered by Guangzhou General Hospital of the Chinese People’s Liberation Army (PLA). The placenta MRA, legs MRA, and the thorax CTA data sets were gained from Shenzhen No.2 People’s Hospital. These clinical data sets were shown in Fig. 5, and the sizes and elements spacing of these data were listed in Table 2.

**Table 2:** Data sizes and elements spacing of the clinical data

	Data sizes (voxels)	Elements spacing (mm)
Brain MRA data	561 × 561 × 361	0.50 × 0.50 × 0.50
Placenta MRA data	512 × 512 × 293	0.40 × 0.40 × 0.70
Legs MRA data	330 × 384 × 66	1.17 × 1.17 × 1.30
Thorax CTA data	512 × 512 × 338	0.36 × 0.36 × 0.4

**Fig. 6:**
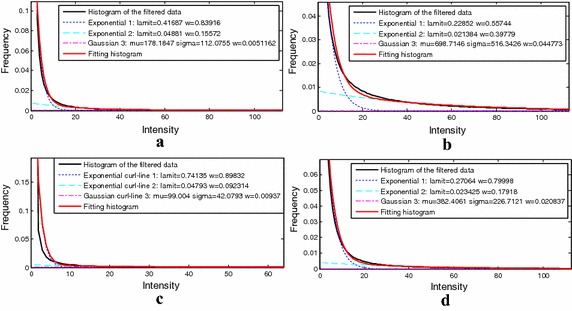
The mixture model fitting results from the angiographic image data of the anatomical parts of the **a** brain, **b** placenta, **c** legs, and **d** thorax

The images of different organs from various imaging equipment exhibited different statistical characteristics. After using the vessel enhancement algorithm, the histograms of the filtered data of these clinical data obtained a new relatively consistent distribution. For the filtered data, the vessel class was modelled by a Gaussian distribution, and the background class was modelled by two Exponential distributions. The results of distribution curves fitting are shown in Fig. 6.

**Fig. 7:**
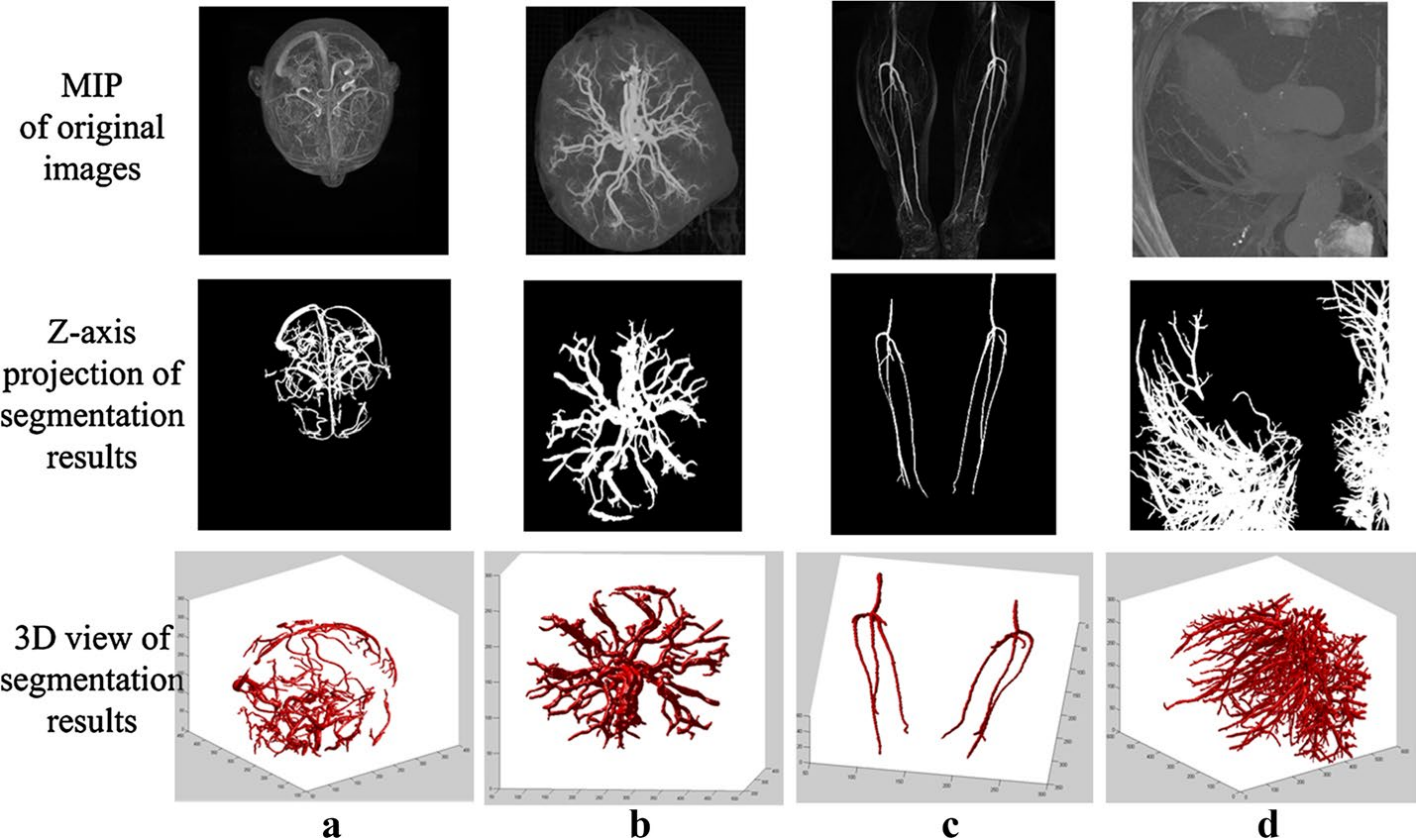
The MIP of the original images compared to the Z-axis projections and 3D views of the segmentation results from the angiographic image of the anatomical parts of the **a** brain, **b** placenta, **c** legs, and **d** thorax

For clinical validation with respect to the real angiographic data, the Z-axis projections as well as the 3D views of the segmentation results were compared to the corresponding maximal intensity projection (MIP) of the original images, as suggested by clinical experts on cardio- and cerebro-vascular diseases. Parts of the results are given in Fig. 7.

### Experiment of comparison tests

As an amendment to above-mentioned quantitative and clinical validation experiments, comparison tests were conducted against the traditional methods. Since our study was mainly based on statistical models, three popular model-based segmentation methods were chose for the tests. The three algorithms were methods of Wilson et al. [20], Gao et al. [22], and Zhou et al. [23]. The mixture models used in the proposed method and the three traditional ones were different from each other, as shown in Table 3. Besides, the proposed mixture model was used to fit the filtered data, and the mixture models of the other three were used to fit the original data. To get the best segmentation results from each of the traditional methods, brain MRA data was used for the tests according to their application ranges. During the validation, the three traditional methods were implemented using models specified in the original papers and parameters as best as possible.

**Table 3:** Mixture models used in the four methods

Method	Class		
	Background	Vessel	Modelled data/morphology
Wilson et al.	Two Gaussian distributions	A uniform distribution	Brain/TOF-MRA
Gao et al.	A Rayleigh and several Gaussian distributions	A Gaussian distribution	Brain/TOF-MRA
Zhou et al.	A Rayleigh and two Gaussian distributions	A Gaussian distribution	Brain/TOF-MRA
Proposed	Two Exponential distributions	A Gaussian distribution	All anatomical parts, all the angiography

**Fig. 8:**
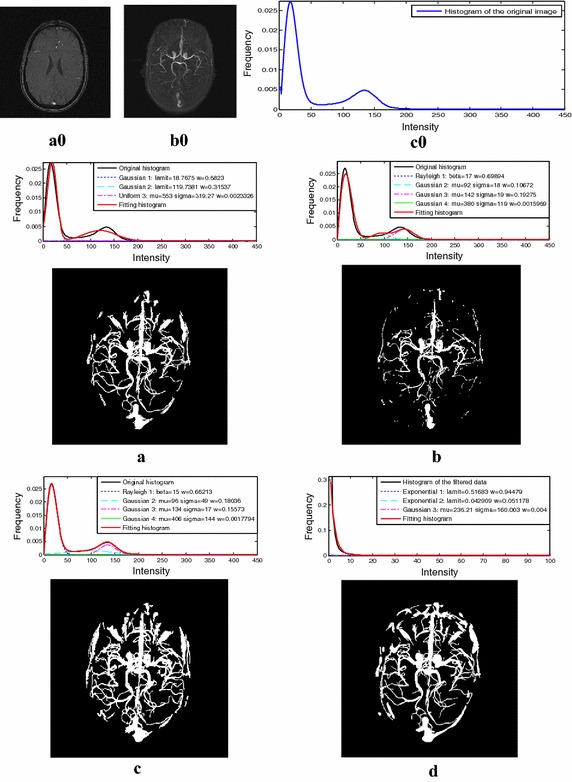
Segmentation results of one online MRA data set. **a0** One slice of the MRA data. **b0** MIP of the MRA data. **c0** Histogram curve of the MRA data. **a** Method of Wilson et al. **b** Method of Gao et al. **c** Method of Zhou et al. **d** The proposed method

The online brain MRA data-sets (http://www.insight-journal.org/midas/community/view/21) have previously been used by many researchers to test their vessel segmentation methods. They were acquired at the element spacing of 0.5 × 0.5 × 0.8 mm^3^ with the data volumes of 448 × 448 × 128 voxels. Comparison tests were conducted on the ten data-sets, and the experimental results of a single MRA data from the four segmentation methods are displayed in Fig. 8.

**Fig. 9:**
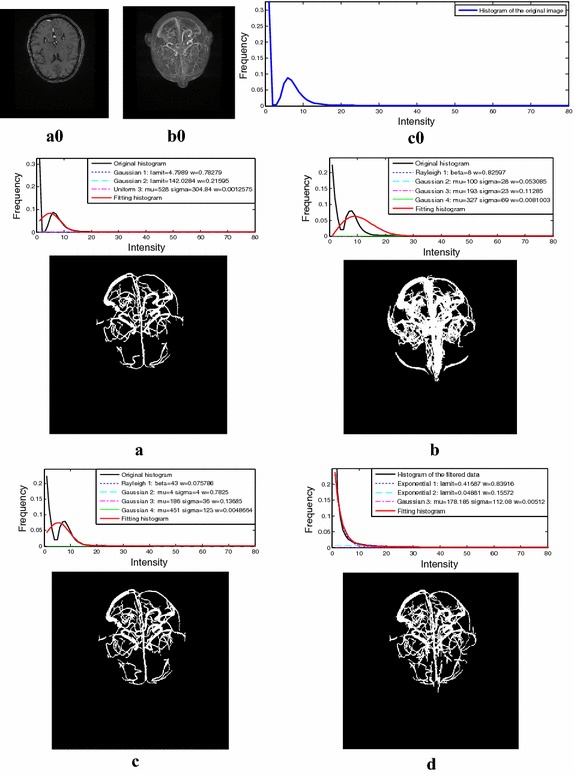
Segmentation results of a single MRA data set from the hospital. **a0** One slice of the MRA data. **b0** MIP of the MRA data. **c0** Histogram curve of the MRA image. **a** Method of Wilson et al. **b** Method of Gao et al. **c** Method of Zhou et al. **d** The proposed method

To further verify the effectiveness of the proposed method, comparison tests were also conducted on some data sets offered by the aforementioned Guangzhou General Hospital of the Chinese PLA. The image acquisition parameters of these data together with those of the online brain MRA data sets were listed in Table 4. Because of uneven contrast media and bias field, these MRA data sets exhibited a poor image quality. One brain MRA data set and its histogram curve are shown in Fig. 9, from which we can see this kind of data hold a different statistical property.

**Table 4:** Data sizes and elements spacing of the images in experiment 3

	Data sizes (voxels)	Elements spacing (mm)
The online brain MRA data	448 × 448 × 128	0.50 × 0.50 × 0.80
Brain MRA data from the hospital	561 × 561 × 361	0.50 × 0.50 × 0.50

## Results and discussions

In this paper, multi-scale filtering algorithm was firstly used for vessel enhancement and noise suppression before the statistical based classification, where filter parameters settings of *α*, *β*, *c* were same for all the data-sets. Suggested by Frangi’s method [6], a compromise value of *α* and *β* equal to 0.5 would make multi-scale filtering algorithm to differentiate tubular-like structures from plate-, and blob-like ones; while the parameter *c* depended on the grey-scale range of the image and was set as half the value of the maximum Hessian norm. The filtered data from multi-modality angiographic images turns to a relatively consistent statistical distribution after the vessel enhancement algorithm. The following statistical model used two Exponential distributions and one Gaussian distribution to fitting the relatively consistent statistical distribution, which classified the vessels through adjusting model components to fit a certain histogram curve of the filtered data. To sum up, as a flexible segmentation method, it performs well in vessel enhance and histogram fitting for the filtered data and is robust to various angiographic images from different human organs and different imaging equipment.

For phantom validation, the method performed well for images despite various signal noises. The tubular object was enhanced while background noise was suppressed effectively. The accuracy of the proposed method was tested by several 3D phantoms. The phantoms were built to simulate the real angiographic images where different noises were added to simulate the background voxels. Compared to the known ground truth, the mixture model efficiently fitted the histogram curves of both phantoms with the segmentation error ratios being 0.21 and 0.11%, and the DSCs being 94.74 and 96.94%. See Table 1, which strongly confirmed the segmentation quality.

For clinical validation, the proposed method was tested on multi-modality angiographic images from different human organs. The mixture model in our method fitted well in each histogram curve of the filtered data. Comparing with the MIP from the original images and the segmentation results, the complete vessel trees were extracted while lesser non-vessels and background were falsely classified as vessel. The results were satisfactory according to the suggestion of clinical experts. Parts of the results are given in Fig. 7.

In comparison tests, the results from traditional statistical based methods and the proposed method were presented. Experimental results of a single MRA data from the four segmentation methods are displayed in Fig. 8. The mixture model built by Zhou et al. obtained the best fitting performance among the three traditional methods according to the histogram fitting performance and the Z-axis projection of these segmentation results. In their method, a Gaussian distribution was used to model the vessel class, while a Rayleigh distribution and two Gaussian distributions were used to model the background class. At the same time, the mixture model built in this paper fitted well for the histogram of the filtered data, and more small vessels were marked out which can been seen from the Z-axis projection of its segmentation result. From Figs. 8 and 9 we can see the mixture model built in Zhou’s method exhibited a good performance in histogram fitting for the online MRA data, but not for the second MRA data which contains a different statistical characteristic. In contrast, the mixture model generated from our method fitted well for both filtered data whose histograms had a relatively consistent statistical property. As a result, the proposed method demonstrated stronger robustness.

**Fig. 10:**
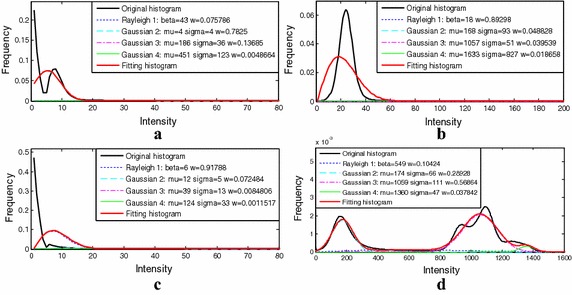
The model fitting results of the clinical data described in experiment 2 by the method of Zhou et al. **a** Brain. **b** Placenta. **c** Legs. **d** Thorax

For fitting the histogram curves of the original images, and the mixture models developed by Zhou et al. exhibited the best fitting performance comparing with the traditional models. However, different angiographic images correspond to different histogram curves. The four clinical data sets from different organs that were described in the part of clinical validation experiment were segmented by Zhou et al.’s method, and the model fitting results are shown in Fig. 10. As a result, the traditional method is not available to classify vessels from angiographic images which hold a different statistical characteristic. In other words, the traditional methods are limited in multi-modality application.

On the basis of Zhou et al.’s work, our research focuses on vessel segmentation from various angiographic images. In our method, different angiographic images were firstly processed by multi-scale filtering algorithm. As shown in Fig. 2, the filtered data sets achieved a relatively consistent distribution. For the vessel class, a Gaussian distribution was built to model its intensity distribution, while two Exponential distributions for the background class. As is shown in Figs. 4, 6, 8, and 9, the above mixture model demonstrated good model fitting performance for the different filtered data sets. As a result, the only known limitation of our approach is that, it is not available for the angiographic images when multi-scale filtering is not fitted for their vessel enhancement. However, the proposed method still exhibits important clinical application value towards various angiographic images, and this kind of exploration ideas is very meaningful in other scientific researches. With the aim of contributing to the field of interventional surgery, our future works are primarily focused on vascular path planning and 3D augmented reality.

## Conclusions

In this paper, a flexible segmentation method has been proposed for vessel extraction. In the first step, the original image data was filtered with multi-scale filtering algorithm. The filtered data obtained a relatively consistent statistical property, and was modelled with a mixture model which consisted of two Exponential distributions and one Gaussian distribution. The EM algorithm was used for parameter estimation, while the MAP–MRF algorithm was used in the Bayesian classification. The proposed method has been tested on phantoms as well as the various modality angiographic images from MRA and CTA data from different imaging equipment. Each histogram of the filtered data from these multi-modality angiographic images could be modelled by relatively consistent mixture probability distributions, and the mixture model displayed a good fitting performance. The segmentation error ratios of the phantoms were less than 0.3% and the DSCs were above 94%. The results of the vessel segmentation procedures have indicated that the method in this paper was accurate and robust for vessel segmentation from multi-modality angiographic images.

## Abbreviations

EM: expectation maximization
3D: three-dimensional
MAP–MRF: maximum a posteriori and Markov random field
MAP: maximum a posteriori
MRF: Markov random field
CTA: computed tomography angiography
MRA: magnetic resonance angiography
MLL: multi-level logistic
MP-NBS: multi-pattern neighbourhood system
ICM: iterated conditional modes
PLA: People’s Liberation Army
MIP: maximal intensity projection
